# The Duration and Magnitude of Postdischarge Venous Thromboembolism Following Colectomy

**DOI:** 10.1097/SLA.0000000000005563

**Published:** 2022-07-19

**Authors:** Christopher A. Lewis-Lloyd, David J. Humes, Joe West, Oliver Peacock, Colin J. Crooks

**Affiliations:** * Gastrointestinal Surgery, National Institute for Health Research (NIHR) Nottingham Biomedical Research Centre (BRC), Nottingham University Hospitals NHS Trust and the University of Nottingham, School of Medicine, Queen’s Medical Centre, Nottingham, UK; † Gastrointestinal and Liver Theme, National Institute for Health Research (NIHR) Nottingham Biomedical Research Centre (BRC), Nottingham University Hospitals NHS Trust and the University of Nottingham, School of Medicine, Queen’s Medical Centre, Nottingham, UK; ‡ Lifespan and Population Health, University of Nottingham, School of Medicine, Nottingham, UK; § Department of Colon and Rectal Surgery, The University of Texas MD Anderson Cancer Center, Houston, TX

**Keywords:** colorectal surgery, colorectal cancer, benign colorectal disease, venous thromboembolism, postoperative complication

## Abstract

To assess the impact of current guidelines by reporting weekly postoperative postdischarge venous thromboembolism (VTE) rates.

**Summary Background Data:**

Disparity exists between the postoperative thromboprophylaxis duration colectomy patients receive based on surgical indication, where malignant resections routinely receive 28 days extended thromboprophylaxis into the postdischarge period and benign resections do not.

**Methods:**

English national cohort study of colectomy patients between 2010 and 2019 using linked primary (Clinical Practice Research Datalink) and secondary (Hospital Episode Statistics) care data. Stratified by admission type and surgical indication, absolute incidence rates (IRs) per 1000 person-years and adjusted incidence rate ratios (aIRRs) for postdischarge VTE were calculated for the first 4 weeks following resection and postdischarge VTE IRs for each postoperative week to 12 weeks postoperative.

**Results:**

Of 104,744 patients, 663 (0.63%) developed postdischarge VTE within 12 weeks after colectomy. Postdischarge VTE IRs per 1000 person-years for the first 4 weeks postoperative were low following elective resections [benign: 20.66, 95% confidence interval (CI): 13.73–31.08; malignant: 28.95, 95% CI: 23.09–36.31] and higher following emergency resections (benign: 47.31, 95% CI: 34.43–65.02; malignant: 107.18, 95% CI: 78.62–146.12). Compared with elective malignant resections, there was no difference in postdischarge VTE risk within 4 weeks following elective benign colectomy (aIRR=0.92, 95% CI: 0.56–1.50). However, postdischarge VTE risks within 4 weeks following emergency resections were significantly greater for benign (aIRR=1.89, 95% CI: 1.22–2.94) and malignant (aIRR=3.13, 95% CI: 2.06–4.76) indications compared with elective malignant colectomy.

**Conclusions:**

Postdischarge VTE risk within 4 weeks of colectomy is ∼2-fold greater following emergency benign compared with elective malignant resections, suggesting emergency benign colectomy patients may benefit from extended VTE prophylaxis.

Colectomy is the second most common major inpatient abdominal operation globally, comprising 2.5% of all surgical procedures performed in the United States each year.^1^,^2^ However, colorectal resections have one of the highest abdominal postoperative risks of venous thromboembolism (VTE), an avoidable and potentially fatal major postoperative complication.^3^,^4^ This risk is especially elevated post emergency and colorectal cancer (CRC) surgery with over a third of VTEs occurring postdischarge.^5^–^7^

To prevent postoperative VTE, international guidance strongly recommends combined mechanical and pharmacological VTE prophylaxis as an inpatient, or if discharged within the first week a minimum 7 days postoperative pharmacological VTE prophylaxis is required following benign colectomies, and for 4 weeks post-CRC resection.^8^,^9^ However, evidence used for these recommendations is of moderate to low quality, predominantly analyzed open elective operations only and no studies have examined weekly VTE rates following colectomy.^10^ Furthermore, changes in operative management might have altered postcolectomy VTE risk, for example, the uptake of minimally invasive surgery,^11^,^12^ and additional groups that some now consider are at higher risk of postoperative VTE, such as those undergoing emergency benign or inflammatory bowel disease (IBD) resections, might now warrant extended VTE prophylaxis.^7^,^13^–^17^

Therefore, contemporary stratified VTE rates following colorectal resection are needed to accurately define the duration and magnitude of postoperative VTE risk within this population to aid guidance on VTE prophylaxis length. Thus, this study describes detailed stratified postdischarge VTE rates in different discharge time risk periods following colectomy to inform extended VTE prophylaxis guidelines.

## METHODS

The study was approved by the Independent Scientific Advisory Committee approval board (Protocol 19_180RA3) and followed Strengthening the Reporting of Observational Studies in Epidemiology (STROBE) guidelines (Supplemental Digital Content 1, http://links.lww.com/SLA/E5).

### Data Sources

This study utilized 3 validated and linked healthcare databases previously described.^18^,^19^ The Clinical Practice Research Datalink (CPRD), comprising CPRD GOLD and Aurum databases, contains primary care prescription and diagnostic data for ∼60 million patients of the United Kingdom (UK) general population, with 16 million actively contributing data.^20^ Since 1989 Hospital Episode Statistics (HES) has compiled detailed records for each episode of admitted patient care delivered in England, either by the National Health Service (NHS) or commissioned by the NHS within the independent sector.^21^ HES patient records are coded using a combination of the International Statistical Classification of Diseases and Related Health Problems 10th Revision (ICD-10) codes for discharge diagnoses and the Office of Population, Censuses and Surveys Classification of Surgical Operations and Procedures version 4 (OPCS-4) codes for detailing procedures relating to an admission. These data have been shown to be demographically equivalent to data from the UK population having been compared with Office for National Statistics data.^22^,^23^

### Cohort

The cohort of patients undergoing colectomy between the years 2010 and 2019 were identified using OPCS-4 codes (eMethods, Supplemental Digital Content 2, http://links.lww.com/SLA/E4) from HES data that was linked to CPRD GOLD and Aurum general practitioner practices. Within the CPRD databases, data quality can be assessed using 2 sets of metrics, acceptability and the up to standard date. Data is only considered acceptable if specific quality measures are met including valid age, sex, accuracy of recorded patient events and registration status. The up to standard date focuses on the accuracy of data continuity recorded by individual primary care practices for patients and is the latest date at which these practices met the minimum quality criteria outlined by the CPRD.^24^ Therefore, patients were only included within the validated cohort if data collection time periods for both primary and secondary care databases coincided, and their data classed as up to research standard and acceptable.^25^

**FIGURE 1:**
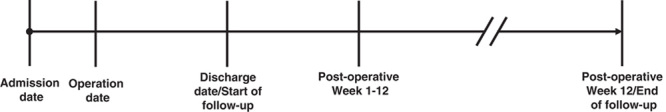
Summary and example timeline of patient follow-up period. Person-time and postdischarge follow-up started the day after the discharge date and ended 12 weeks post the operation date. VTE events occurring in this period were classed as postoperative postdischarge VTEs.

Completely endoscopic operations, those confined to the anal canal, and patients aged less than 18 years old were excluded. Patients identified as having a VTE event before colectomy were also excluded due to their inherently increased risk of VTE.^26^ Person-time and postdischarge follow-up started the day after the date of hospital discharge. All follow-up lasted until earliest date of VTE event, death, change to a nonparticipating general practice or 12 weeks from the date of operation (Fig. 1). Twelve weeks postoperative follow-up was chosen from previous literature.^27^

### Exposures

Admission type, surgical indication and operative technique were the main exposures of interest and selected from previous literature.^7^ Admission type was defined as elective or emergency based on the admission classification recorded for the surgical procedure in HES data. Surgical indication was defined as benign or malignant. A benign indication was defined using ICD-10 discharge codes, including IBD, diverticular disease and other (eMethods, Supplemental Digital Content 2, http://links.lww.com/SLA/E4). A malignant indication was defined within the CPRD and HES data using relevant ICD-10 codes for CRC (C18–C20, excluding C18⋅1; Appendix). Operative technique was classified into open and minimally invasive surgery. Minimally invasive technique was defined as laparoscopic or robotic surgery using OPCS-4 codes (Y50.8, Y57.1, and Y75.2) and (Y75.3), respectively. Procedures that began using a minimally invasive approach were classed as minimally invasive surgery. Age at time of operation was categorized into less than 60, 60–69, 70–79, and ≥ to 80 years and as a binary variable, ≤ 60 and above 60 years. Comorbidity was classified using the Charlson index before surgical admission and determined from CPRD and HES data and categorized into 0, 1, and ≥2 as well the binary variable 0 or ≥1.^28^ These binary variables were based on the National Institute for Health and Care Excellence (NICE) VTE risk assessment tool score groupings.^8^ Sex was defined from HES data as either male or female. Ethnicity was determined from HES and categorized as White, other or unknown. Postoperative hospital length of stay was defined from HES as the time from colectomy operation to discharge date and classed as a binary variable, ≤7 or >7 days postoperation.

National English NICE guidance on postoperative VTE prevention during the cohort time frame stated that those undergoing abdominal surgery at risk of VTE should receive combined mechanical and chemical VTE prophylaxis during admission and those undergoing cancer surgery should receive extended 28 days of postoperative VTE chemoprophylaxis.^8^ Therefore, within a population-based cohort from a nationally organized health service, those undergoing benign resections were deemed to have received inpatient but not extended or postdischarge VTE prophylaxis while those undergoing malignant resections were deemed to have received inpatient and a total of 28 days extended VTE prophylaxis and where applicable, prophylaxis into the postdischarge period.

### Outcomes

The primary outcome was a postoperative postdischarge VTE event diagnosis defined from medical and ICD-10 codes in the linked CPRD and HES datasets. Postdischarge VTE was defined as a VTE event occurring after the HES colectomy discharge date. VTE events were only considered valid if supported by either a prescription for any anticoagulant medication from the British National Formula licensed to treat a VTE in the UK or other evidence of treatment within an anticoagulation clinic (eg, a medical code) between 15 days before and 90 days after VTE diagnosis, or a date of death within 30 days of the event. Furthermore, VTE as an underlying cause of death was included as evidence of VTE diagnosis. Only the first confirmed VTE episode was incorporated within the analysis with further follow-up time censored. This definition using primary care data has been formally validated and published, specifically validating a VTE diagnosis against individual patient records with a positive predictive value of 84%^29^ and extensively used in previous studies.^7^

### Statistical Analysis

Cohort demographics were presented as proportions and stratified by admission type and operative technique. Absolute incidence rates (IRs) of VTE were calculated per 1000 person‐years with 95% confidence intervals (CIs).

Postdischarge VTE rates for each week from the date of operation up to 12 weeks following colectomy were calculated and stratified *a priori* by admission type and surgical indication then operative technique.^7^–^9^ However, it was anticipated numbers undergoing emergency minimally invasive colectomies would be small preventing stratification by operative technique.^30^,^31^

Postdischarge VTE rates and adjusted incidence rate ratios (aIRRs) for linear trend, adjusted for age, sex, comorbidity, and operative technique, were calculated using a Poisson regression model for the first 4 weeks following colectomy and stratified *a priori* by admission type and surgical indication.^7^–^9^ To assess the impact of IBD resections on overall benign colectomy postdischarge VTE risk, a sensitivity analysis of the multivariable model was performed with benign surgical indications further stratified into “benign other” and IBD conditions.

In addition, where numbers were sufficient, postdischarge VTE rates were assessed by other VTE risk factors; age, less than or equal to 60 or above 60 years old, comorbidity, Charlson score 0 or ≥1, and postoperative length of stay, ≤7 or >7 days, stratified by admission type for each week following the date of surgery up to postoperative week 12.^6^,^7^

All data management and analyses were performed using Stata SE, version 16.1 (StataCorp LLC, College Station, TX).

## RESULTS

### Cohort Demographics

Of 104,744 patients undergoing colectomy included in the cohort, 663 (0.63%) developed a postdischarge VTE within 12 weeks following resection. Over half of elective colectomies were performed using a minimally invasive approach (58.88%) with an increase in proportion over the study period from 41.77% in 2010 to 73.36% by 2019. The overall conversion from minimally invasive to open colectomy was 7.99%, and 8.75% for elective and 6.40% for emergency resections, respectively. Elective patients were older (60 years and older 67.16%), underwent malignant resections (69.89%) and had ≥1 significant comorbidity (83.95%). Most emergency colectomies were performed using an open approach (82.49%) with an increase in minimally invasive surgery over the study period from 10.20% in 2010 to 26.16% by 2019 and underwent resections for benign disease (69.28%). Over half of emergency patients had ≥1 significant comorbidity (63.29%). Median postoperative length of stay following colectomy was 7 days [interquartile range (IQR): 5–11 days] for elective benign, 7 days (IQR: 5–10 days) for elective malignant, 11 days (IQR: 7–20 days) for emergency benign and 11 days (IQR: 7–19 days) for emergency malignant resections. Overall, 49.32% of patients were in hospital for ≤7 days postoperation (Table 1).

**TABLE 1:** Demographics of Colectomy Cohort, by Admission Type and Operative Technique

	N=104,744 [n (%)]			
	Emergency (N=34,083)		Elective (N=70,661)	
	Open (n=28,115)	Minimally Invasive (n=5968)	Open (n=29,059)	Minimally Invasive (n=41,602)
Surgical indication				
Benign	19,205 (68.31)	4406 (73.83)	10,629 (36.58)	10,646 (25.59)
Malignant	8910 (31.69)	1562 (26.17)	18,430 (63.42)	30,956 (74.41)
Age (y)				
<60	10,867 (38.65)	3623 (60.71)	9782 (33.66)	13,421 (32.26)
60–69	5711 (20.31)	849 (14.23)	7326 (25.21)	10,864 (26.11)
70–79	6516 (23.18)	859 (14.39)	7721 (26.57)	11,456 (27.54)
≥80	5021 (17.86)	637 (10.67)	4230 (14.56)	5861 (14.09)
Sex				
Male	13,477 (47.94)	2861 (47.94)	14,827 (51.02)	22,546 (54.19)
Female	14,638 (52.06)	3107 (52.06)	14,232 (48.98)	19,056 (45.81)
Charlson score				
0	9796 (34.84)	2716 (45.51)	4625 (15.92)	6714 (16.14)
1	2562 (9.11)	477 (7.99)	2057 (7.08)	2966 (7.13)
≥2	15,757 (56.04)	2775 (46.50)	22,377 (77.01)	31,922 (76.73)
Ethnicity				
White	25,771 (91.66)	5252 (88.00)	26,871 (92.47)	37,925 (91.16)
Other	1756 (6.25)	558 (9.35)	1725 (5.94)	2656 (6.38)
Unknown	588 (2.09)	158 (2.65)	463 (1.59)	1021 (2.45)
Postoperative length of stay (d)				
≤7	7427 (26.42)	2724 (45.64)	13,257 (45.62)	28,252 (67.91)
>7	20,688 (73.58)	3244 (54.36)	15,802 (54.38)	13,350 (32.09)

Minimally invasive surgery consists of procedure completed either by a laparoscopic or robotic technique.

### Postdischarge VTE Rates by Admission Type and Surgical Indication

#### Weekly Postdischarge VTE Rates

All weekly postdischarge VTE rates were analyzed by admission type and surgical indication. However, only elective malignant procedures had sufficient numbers to allow stratification by operative technique.

#### Elective Colectomy

**FIGURE 2:**
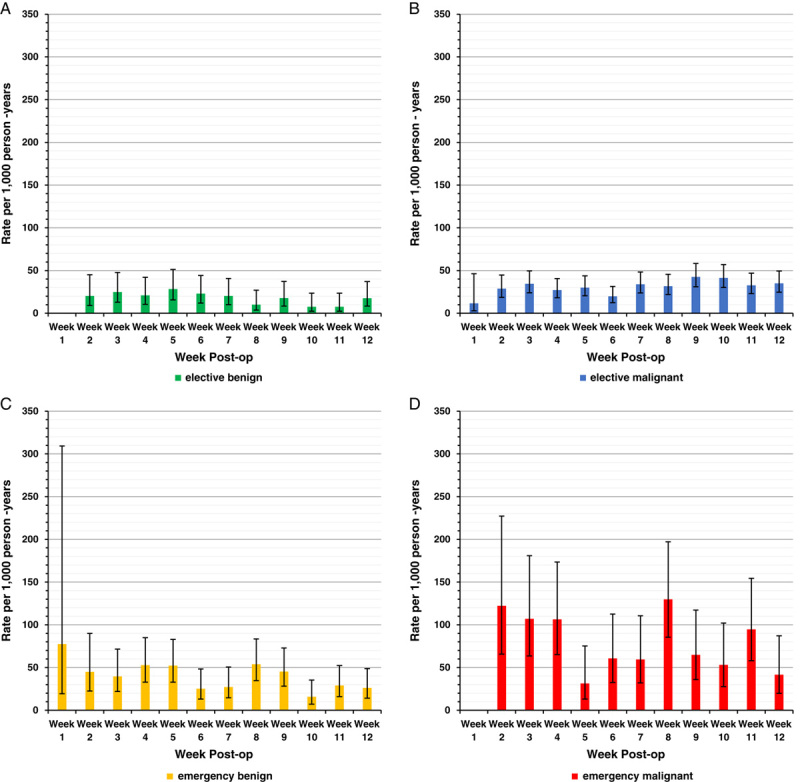
Postdischarge VTE rates by postoperative week stratified by admission type and surgical indication. Elective benign (A), elective malignant (B), emergency benign (C), emergency malignant (D). Data missing within week 1 indicates no postdischarge VTE events occurred within the first postoperative week. Error bars indicate 95% CIs.

**FIGURE 3:**
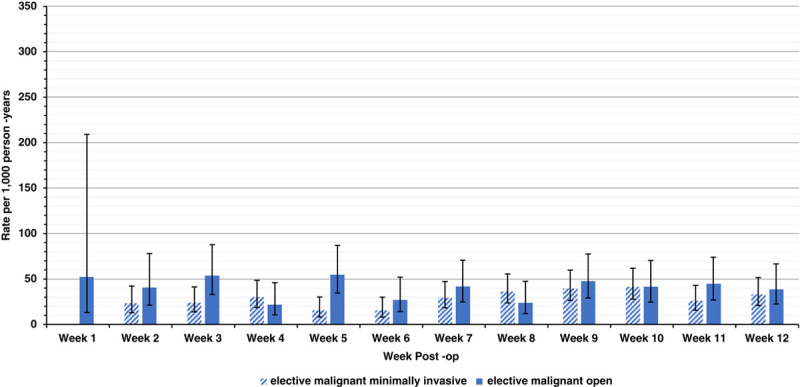
Postdischarge VTE rates by postoperative week following elective malignant colectomy stratified by operative technique. Data missing within week 1 indicates no postdischarge VTE events occurred within the first postoperative week. Error bars indicate 95% CIs.

Following elective benign surgery, all weekly postdischarge VTE rates were <30 per 1000 person-years with no VTE events occurring in discharged patients in the first postoperative week (Fig. 2A). Following elective malignant colectomies, all postdischarge VTE rates were <50 per 1000 person-years (Fig. 2B). However, on stratification by operative technique, following elective malignant open resections postdischarge VTE rates were >50 per 1000 person-years in postoperative weeks 1, 3, and 5 (Fig. 3). All postdischarge VTE rates following elective malignant minimally invasive colectomy were <50 per 1000 person-years with no VTE events in discharged patients in the first postoperative week.

#### Emergency Colectomy

All emergency rates were stratified by surgical indication. However, most emergency colectomies for benign (81.34%) and malignant (85.08%) disease were open resections meaning there were too few minimally invasive emergency colectomies to allow further stratification by operative technique (Table 1). Following emergency benign colectomies, the highest rate of VTE in discharged patients was in the first week following resection (IR=77.36 per 1000 person-years, 95% CI: 19.35–309.34). Then from the second week after the operation postdischarge VTE rates were <50 per 1000 person-years except in postoperative weeks 4, 5, and 8 (Fig. 2C). Following emergency malignant resections, VTE rates in discharged patients were >100 per 1000 person-years from the second to fourth postoperative week, with a further rise in postoperative week 8 (IR=129.79 per 1000 person-years, 95% CI: 85.46–197.11). No VTE events occurred in discharged patients in the first postoperative week, although person-time was negligible (Fig. 2D).

#### Four Weeks Postoperative Postdischarge VTE Rates

Crude VTE rates for the first 4 weeks following colectomy were low for elective resections following both benign, IR 20.66 per 1000 person-years (95% CI: 13.73–31.08), and malignant indications, IR 28.95 per 1000 person-years (95% CI: 23.09–36.31). In contrast, the emergency resection 4 weeks postdischarge VTE rates were higher for both benign, IR 47.31 per 1000 person-years (95% CI: 34.43–65.02), and malignant indications, IR 107.18 per 1000 person-years (95% CI: 78.62–146.12). These differences remained significant after adjusting for age, sex, comorbidity, and operative technique, with a 2- to 3-fold increased risk for emergency benign and malignant resections compared with elective malignant resections (aIRR=1.89, 95% CI: 1.22–2.94 and 3.13, 95% CI: 2.06–4.76, respectively). Elective benign resections did not have an increased risk compared with elective malignant resections (aIRR=0.92, 95% CI: 0.56–1.50) (eTable 1, Supplemental Digital Content 2, http://links.lww.com/SLA/E4).

### Benign Colectomy and IBD

On stratification of benign disease, the 4 weeks postcolectomy postdischarge crude VTE rate following elective IBD resections, IR 11.71 per 1000 person-years (95% CI: 4.39–31.19), was lower than in those undergoing elective “benign other” resections, IR 24.62 per 1000 person-years (95% CI: 15.70–38.60). However, after multivariable adjustment there was no significant difference in postdischarge VTE risk between elective IBD and “benign other” colectomies (aIRR=0.84, 95% CI: 0.28–2.50). In contrast, the 4 weeks postcolectomy postdischarge crude VTE rate following emergency IBD resections, IR 62.49 per 1000 person-years (95% CI: 37.01–105.52), was higher than in those undergoing emergency “benign other” resections, IR 41.44 per 1000 person-years (95% CI: 27.78–61.83). After multivariable adjustment, there was a >2.5-fold increased risk of postdischarge VTE in emergency IBD compared with “benign other” colectomies (aIRR=2.54, 95% CI: 1.29–5.02).

### Weekly Postdischarge VTE Rates by Age, Comorbidity, and Postoperative Length of Stay

Further stratifying postdischarge VTE rates by admission type then age or comorbidity did not change the patterns to postdischarge VTE rates described above (eFigs. 1A, B, Supplemental Digital Content 2, http://links.lww.com/SLA/E4). Stratifying elective patients by their postoperative length of stay showed higher rates following a >7-day admission than in admissions shorter than or equal to a week (eFigs. 3A, B, Supplemental Digital Content 2, http://links.lww.com/SLA/E4). The highest VTE rate in discharged patients after an elective admission lasting >7 days, was in the second week following colectomy (IR=69.73, 95% CI: 41.30–117.74 per 1000 person-years). Following emergency resections, postdischarge VTE rates followed similar patterns previously reported irrespective of postoperative length of stay.

## DISCUSSION

### Overview

Overall, absolute rates of postdischarge VTE following elective benign or malignant colectomy are equivalent within the first 4 weeks post resection with weekly rates below 50 per 1000 person-years, indicating current VTE prophylaxis guidance in these groups of patients is appropriate. Emergency benign colectomies do not routinely receive extended VTE prophylaxis, yet we show that their 4-week postdischarge VTE risk was almost 2-fold greater than those undergoing elective malignant resections that do routinely receive extended VTE prophylaxis. This suggests patients discharged after emergency benign and specifically emergency IBD resections might benefit from a period of extended VTE prophylaxis.

### Strengths and Limitations

The main strength of this large population study is the power to stratify observed postdischarge VTE rates into postoperative weeks by admission type, surgical indication, and known VTE risk factors. This allowed for the quantification of the week VTE rates changed following colectomy within specific subgroups of interest that are at high risk of postoperative VTE. In this study, the VTE definition has previously been validated, capturing both outpatient VTEs from primary as well as those admitted in secondary care. This thereby reduces the surveillance bias that can occur if patients are solely identified while hospitalized.^29^,^32^ Furthermore, by using an unselected population-based cohort study the presented estimates will be generalizable to other populations with universal coverage and developed healthcare systems.

The study population selected underwent colectomy from 2010 onwards, all of which occurred after the introduction of national VTE prophylaxis guidelines.^8^ Although a limitation of this study was the inability to assess the effect of VTE prophylaxis directly, as hospital prescribing at patient level is not currently available, that could lead to misclassification bias of those receiving extended VTE prophylaxis. However, 95% to 96% of NHS acute admissions were risk assessed for VTE according to recent national audit data and therefore for the appropriateness of VTE prophylaxis.^33^,^34^ Therefore, all patient groups in this cohort will have had prophylaxis prescribed in line with the trends in the country as a whole. Although, it would be desirable to have inpatient prescribing, a national cohort large enough to allow detailed stratified results with this data is not currently available. As with any population-based database, there are always concerns surrounding the accuracy of recorded data. However, the databases utilized within this study have been extensively used, previously validated and contain built-in metrics to assure data quality and accuracy, including the use of a validated definition of a VTE outcome.^7^,^18^,^24^,^29^

### Elective Colectomy

After elective benign colectomy, weekly postdischarge VTE rates were relatively low. This was consistent with other studies that reported elective benign colectomies have one of the lowest postcolectomy VTE risks.^7^ Current VTE prophylaxis guidance in this group is therefore appropriate. However, elective patients admitted for >7 days following colectomy had elevated postdischarge VTE rates to week 5 postoperative with previous data showing hospitalization for >1 week has a significant elevating effect on postdischarge VTE rates following colectomy.^6^,^35^ Consequently, if guidance were to be updated, it could be tailored to address certain high-risk elective subgroups, including those with prolonged inpatient stays, that may benefit from further VTE prevention measures such as extended VTE prophylaxis.^36^

Following elective malignant resections, postdischarge VTE rates were also low and similar to elective benign rates. This fits with contemporary reports of VTE rates following CRC and IBD or benign resections being relatively equivalent.^15^,^37^ A conflicting earlier analysis by Humes et al^7^ found the VTE rate postelective malignant colectomy was greater than those with benign disease. However, this may be due to the study period analyzed predating the introduction of extended VTE prophylaxis guidance with most patients operated on via an open approach.^12^,^38^ The low postdischarge VTE rates we report for elective CRC resections could be a consequence of the extended 4 weeks VTE prophylaxis that international guidance already advocates. However, the risk varied by operative technique, with VTE rates in those undergoing elective malignant open resections remaining above 50 per 1000 person-years until postoperative week 5, but patients undergoing minimally invasive colectomy were consistently at very low VTE risks. This latter group might therefore not require extended VTE prophylaxis. Despite Vedovati et al^39^ demonstrating the protective impact of extended VTE prophylaxis within a laparoscopic CRC cohort, debate surrounds the benefit of contemporary extended VTE prophylaxis use in elective CRC resection in conjunction with perioperative advancements including minimally invasive surgery and enhanced recovery protocols.^40^–^42^ Furthermore, within the elective setting those aged 60 years and below, with inpatient durations ≤7 days and or no comorbidities the postdischarge VTE rates were further reduced. Therefore, in these low-risk subgroups extended VTE prophylaxis could potentially be omitted in elective CRC patients, thus reducing morbidity and having cost saving benefits.^43^

### Emergency Colectomy

After emergency benign colectomies, the postdischarge VTE risk within 4 weeks of resection was ∼2-fold greater than those undergoing elective malignant colectomy, with the highest risk in those undergoing IBD resection and the highest weekly postdischarge VTE rate seen in the first postoperative week. This correlates with studies showing emergency benign or IBD colectomy VTE rates are higher than those in the elective setting, including patients undergoing CRC resection that are currently considered higher risk.^7^,^37^,^44^–^46^ Despite this, emergency benign resections that form the majority of emergency colectomy work (69.28%) do not receive extended VTE prophylaxis while elective malignant colectomies do. Therefore, the results of this study suggest emergency benign, and in particular IBD, admissions should receive at least 7 days VTE prophylaxis in line with current guidance and may in fact benefit from extended VTE prophylaxis.^13^,^15^,^17^,^35^

The highest and longest elevated weekly postdischarge VTE rates were observed following emergency malignant resections with a >3-fold increased risk of postdischarge VTE within 4 weeks of colectomy compared those undergoing elective CRC surgery. This is expected and comparable with previous findings within the literature highlighting the increased VTE risk within this population.^7^,^47^,^48^ These high postdischarge VTE rates persisted for the majority of the 12-week follow-up period despite guidelines recommending these patients receive 4 weeks of extended VTE prophylaxis. The relatively high magnitude and duration of VTE rates may be secondary to the combined effects of increasing age, advanced cancer stage and adjuvant chemotherapy, commencing around 6 to 8 weeks postresection, which are known risk factors for VTE particularly within the emergency setting.^48^–^50^ Further perioperative VTE prevention measures, especially in the elderly and other high-risk subgroups, might therefore benefit these patients: For example, increasing emergency minimally invasive colectomy uptake and implementing emergency colorectal enhanced recovery pathways that have been shown to improve postoperative outcomes for elective patients as well as potentially increasing the period of extended VTE prophylaxis.^51^,^52^

## CONCLUSIONS

The postdischarge VTE risk within 4 weeks of surgery following emergency benign resections, particularly in those undergoing IBD resection, is greater than in those undergoing elective malignant colectomy. This is despite emergency benign resections not receiving extended VTE prophylaxis yet comprising the majority of emergency colectomies, indicating they might benefit from extended VTE prophylaxis.

## Supplementary Material

SUPPLEMENTARY MATERIAL
