# Supplementary material for: The Duration and Magnitude of Postdischarge Venous Thromboembolism Following Colectomy
Source: Ann Surg. 2022 Jul 19;276(3):e177–84. doi: 10.1097/SLA.0000000000005563 (PMC9362343; doi:10.1097/SLA.0000000000005563)
Supplement: SUPPLEMENTARY MATERIAL [file sla-276-e177-s001.docx]

# **SUPPLEMENTARY DIGITAL CONTENT**

**eMethods:** OPCS and ICD codes used to identify colectomy, inflammatory bowel disease and diverticular disease.

**eFigure 1: Post-discharge venous thromboembolism rates by post-operative week stratified by admission type and age**

(A) Elective (B) Emergency

Data missing within Week 1 indicates no post-discharge venous thromboembolism events occurred within the first post-operative week.

Error bars indicate 95% confidence intervals.

**eFigure 2: Post-discharge venous thromboembolism rates by post-operative week stratified by admission type and co-morbidity**

(A) Elective (B) Emergency

Data missing within Week 1 indicates no post-discharge venous thromboembolism events occurred within the first post-operative week.

Error bars indicate 95% confidence intervals.

**eFigure 3: Post-discharge venous thromboembolism rates by post-operative week stratified by admission type and post-operative length of stay**

(A) Elective (B) Emergency

Data missing within Week 1 indicates no post-discharge venous thromboembolism events occurred within the first post-operative week.

Error bars indicate 95% confidence intervals.

**eTable 1: 4-Weekly rates of post-operative post-discharge venous thromboembolism rates by admission type and surgical indication**

^a^Adjusted for age, sex, Charlson score and operative technique.

## *eMethods*

OPCS and ICD codes used to identify colectomy, inflammatory bowel disease and diverticular disease

**Colectomy codes**

H041, H042, H043, H048, H049, H051, H052, H053, H058, H059, H061, H062, H063, H064, H068, H069, H071, H072, H073, H074, H078, H079, H081, H082, H083, H084, H085, H088, H089, H091, H092, H093, H094, H095, H098, H099, H101, H102, H103, H104, H105, H108, H109, H111, H112, H113, H114, H115, H118, H119, H291, H292, H293, H294, H298, H299, H331, H332, H333, H334, H335, H336, H337, H338, H339

**Inflammatory bowel disease codes**

K50, K500, K501, K508, K509, K51, K510, K512, K513, K514, K515, K518, K519, K520, K521, K522, K523, K528, K529

**Diverticular disease codes**

K57, K570, K571, K572, K573, K574, K575, K578, K579


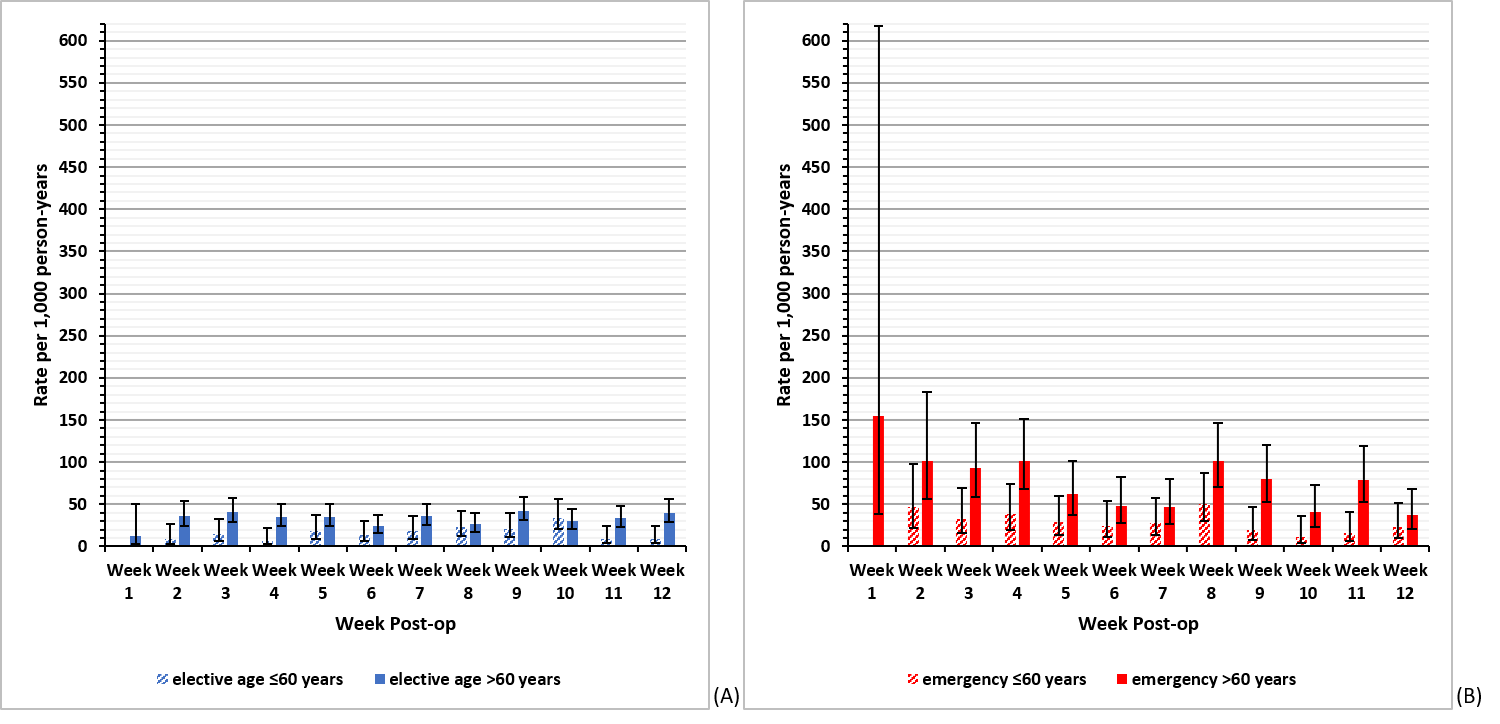


**eFigure 1: Post-discharge venous thromboembolism rates by post-operative week stratified by admission type and age**

(A) Elective (B) Emergency

Data missing within Week 1 indicates no post-discharge venous thromboembolism events occurred within the first post-operative week.

Error bars indicate 95% confidence intervals.


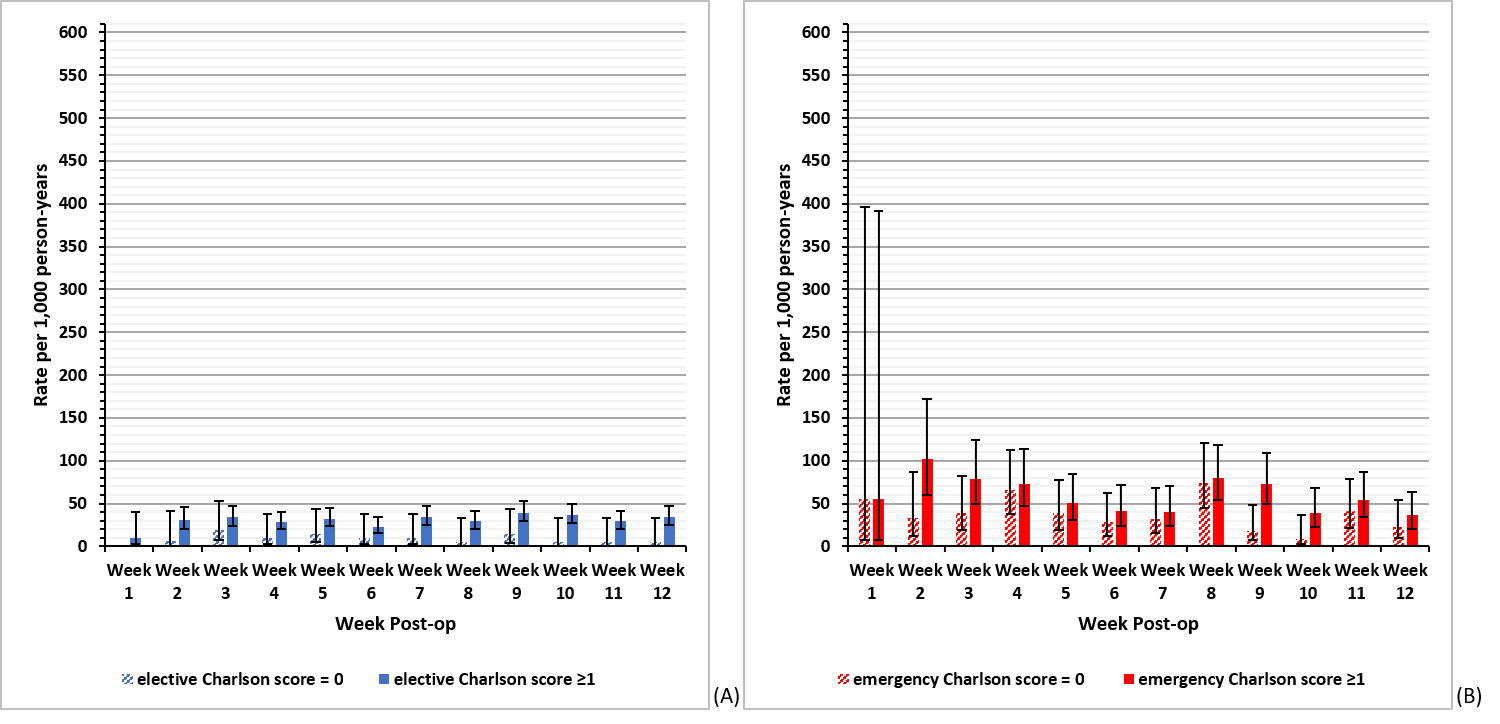


**eFigure 2: Post-discharge venous thromboembolism rates by post-operative week stratified by admission type and co-morbidity**

(A) Elective (B) Emergency

Data missing within Week 1 indicates no post-discharge venous thromboembolism events occurred within the first post-operative week.

Error bars indicate 95% confidence intervals.


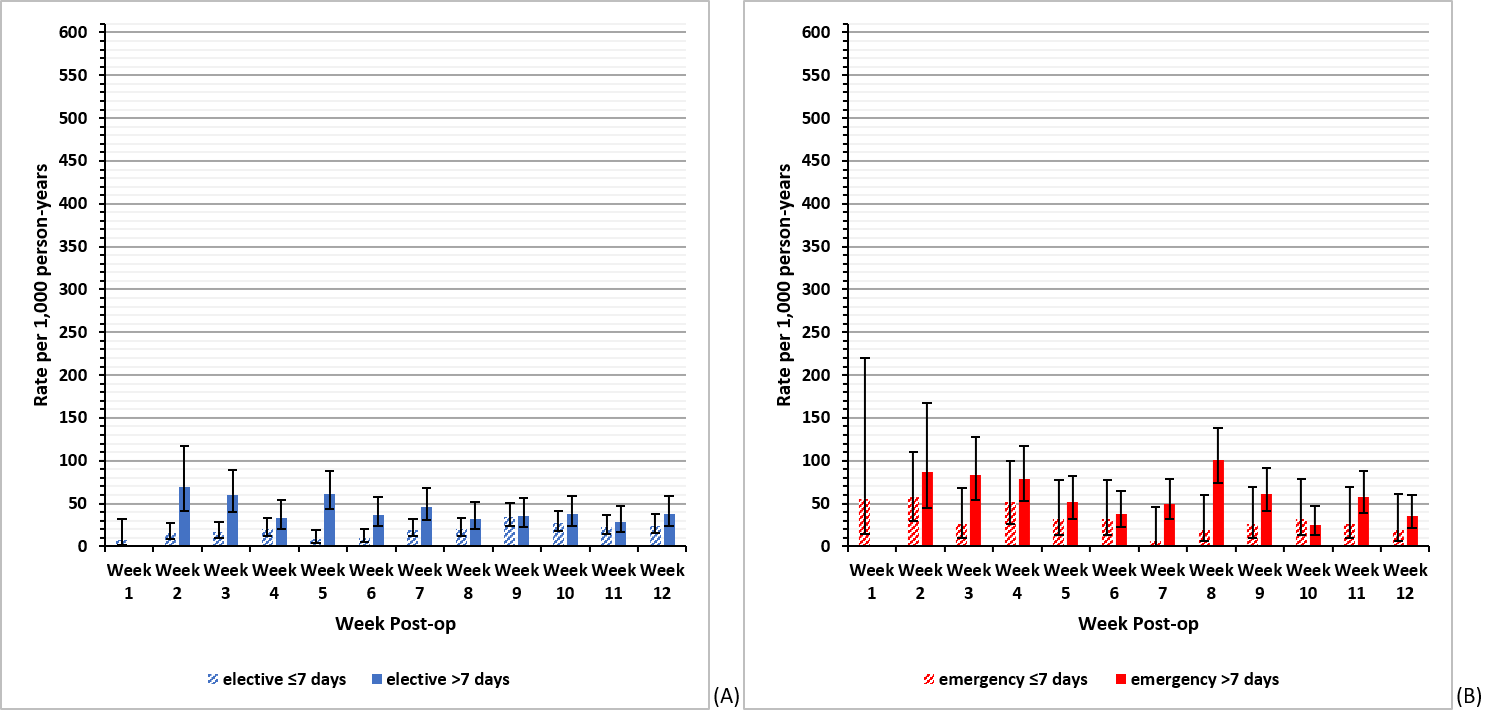


**eFigure 3: Post-discharge venous thromboembolism rates by post-operative week stratified by admission type and post-operative length of stay**

(A) Elective (B) Emergency

Data missing within Week 1 indicates no post-discharge venous thromboembolism events occurred within the first post-operative week.

Error bars indicate 95% confidence intervals.

|  | **Event No.** | **Person-years** | **Rate per 1,000 person-years** | | | **Unadjusted Incidence Rate Ratio** | | | **Adjusted Incidence Rate Ratio^a^** | | |
| --- | --- | --- | --- | --- | --- | --- | --- | --- | --- | --- | --- |
| **Elective Malignant** | 75 | 2.59 | **28.95** | 23.09 | 36.31 | **1.00** | (Reference) | | **1.00** | (Reference) | |
| **Elective Benign** | 23 | 1.11 | **20.66** | 13.73 | 31.08 | **0.71** | 0.45 | 1.14 | **0.92** | 0.56 | 1.50 |
| **Emergency Benign** | 38 | 0.80 | **47.31** | 34.43 | 65.02 | **1.63** | 1.11 | 2.41 | **1.89** | 1.22 | 2.94 |
| **Emergency Malignant** | 40 | 0.37 | **107.18** | 78.62 | 146.12 | **3.70** | 2.52 | 5.43 | **3.13** | 2.06 | 4.76 |

**eTable 1: 4-Weekly rates of post-operative post-discharge venous thromboembolism rates by admission type and surgical indication**

^a^Adjusted for age, sex, Charlson score and operative technique.
